# Little genomic support for Cyclophilin A-matrix metalloproteinase-9 pathway as a therapeutic target for cognitive impairment in *APOE4* carriers

**DOI:** 10.1038/s41598-022-05225-8

**Published:** 2022-01-20

**Authors:** Emma L. Anderson, Dylan M. Williams, Venexia M. Walker, Neil M. Davies

**Affiliations:** 1 Medical Research Council Integrative Epidemiology Unit, University of Bristol, Bristol, BS8 2BN UK; 2 Population Health Sciences, Bristol Medical School, University of Bristol, Barley House, Oakfield Grove, Clifton, Bristol, BS8 2BN UK; 3 MRC Unit for Lifelong Health and Ageing at UCL, University College London, London, UK; 4 Department of Public Health and Nursing, K.G. Jebsen Center for Genetic Epidemiology, NTNU, Norwegian University of Science and Technology, Trondheim, Norway

**Keywords:** Epidemiology, Alzheimer's disease

## Abstract

Therapeutic targets for halting the progression of Alzheimer’s disease pathology are lacking. Recent evidence suggests that APOE4, but not APOE3, activates the Cyclophilin-A matrix metalloproteinase-9 (CypA-MMP9) pathway, leading to an accelerated breakdown of the blood–brain barrier (BBB) and thereby causing neuronal and synaptic dysfunction. Furthermore, blockade of the CypA-MMP9 pathway in APOE4 knock-in mice restores BBB integrity and subsequently normalizes neuronal and synaptic function. Thus, CypA has been suggested as a potential target for treating APOE4 mediated neurovascular injury and the resulting neuronal dysfunction and degeneration. The odds of drug targets passing through clinical trials are greatly increased if they are supported by genomic evidence. We found little evidence to suggest that CypA or MMP9 affects the risk of Alzheimer’s disease or cognitive impairment using two-sample Mendelian randomization and polygenic risk score analysis in humans. This casts doubt on whether they are likely to represent effective drug targets for cognitive impairment in human APOE4 carriers.

## Introduction

Therapeutic targets for slowing or halting the progression of Alzheimer’s disease pathology are lacking. There has recently been considerable interest in the proinflammatory Cyclophilin A–matrix metalloproteinase-9 (CypA–MMP9) pathway as a potential drug target for the treatment of Alzheimer’s disease (AD)^1^. It has previously been shown that *APOE4*, but not *APOE3*, activates the CypA–MMP9 pathway, which may lead to accelerated breakdown of the blood brain barrier, and thereby cause neuronal and synaptic dysfunction. Studies have reported that blockade of the CypA–MMP9 pathway in *APOE4* knock-in mice restores blood brain barrier integrity and subsequently normalises neuronal and synaptic function^2^. Thus, CypA has been suggested as a potential target for treating APOE4-mediated neurovascular injury and the resulting neuronal dysfunction and degeneration. CypA inhibitors, such as cyclosporine, are already licensed for use in humans to treat non-neurological diseases.

The odds of drug targets passing through clinical trials are greatly increased if they are supported by genomic evidence^3^. We aimed to triangulate the existing evidence for the causal effect of CypA and MMP9 on cognitive impairment from experimental studies, with evidence from two-sample Mendelian randomization and polygenic risk score analysis in humans, to examine whether CypA and MMP9 are likely to be effective drug targets for cognitive impairment in human APOE4 carriers.

## Methods

### Mendelian randomization analysis

We used two-sample Mendelian randomization^4^ to examine (i) whether *APOE4* (tagged by the C allele of single nucleotide polymorphism rs429358^5^) has a causal effect on blood-based CypA or MMP9 expression quantitative trait loci (eQTLs) and protein quantitative trait loci (pQTLs), and (ii) whether blood-based CypA and MMP9 eQTLs and pQTLs have a causal effect on the risk of Alzheimer’s disease (AD). Methods for conducting two-sample MR analyses have been published previously^4^. Briefly, two-sample MR provides an estimate of the causal effect of an exposure on an outcome, using independent samples to obtain the gene-exposure and gene-outcome associations, provided three key assumptions hold: (i) genetic variants are robustly associated with the exposure of interest (i.e. replicate in independent samples), (ii) there is no confounding of the causal effect of the genetic variants on the outcome [for example, by population stratification (ref)] and (iii) there are no effects of the genetic variants on the outcome, independent of the exposure (i.e. no horizontal pleiotropy) (ref).

#### Data

We used the largest publicly available blood-based eQTL genome-wide association study (GWAS) meta-analysis (eQTLGen; n = 31,684^6^), pQTL GWAS meta-analysis (n = 3301^7^) and Alzheimer’s disease GWAS meta-analysis (n = 71,880 cases and 383,378 controls^8^). All GWASs used in these analyses were conducted primarily on participants of European ancestry. Ethics approval was obtained by the original studies. F statistics are provided in the results tables. F statistics provide an indication of instrument strength and are a function of how much variance in the trait is explained by the set of genetic instruments being used, the number of genetic instruments being used, and the sample size. F statistics greater than 10 indicate that the analysis is unlikely to suffer from weak instrument bias^9^. All F statistics were above 10.

#### Harmonization procedure and statistical analysis

MR-Base (www.mrbase.org)^10^ was employed to perform all MR analyses. To estimate causal effects of APOE4 on CypA and MMP9 eQTLs and pQTLs, the E4 allele of APOE was tagged by the presence of cysteine at rs429358 (one C for heterozygotes, two for homozygotes). SNP (rs429358)-outcome estimates were extracted from the CypA and MMP9 eQTL and pQTL GWASs and effects of APOE4 on the outcomes are shown in Supplementary Table B below. For causal effects of CypA and MMP9 eQTLs and pQTLs on Alzheimer’s disease, one approximately independent genome-wide significant (p < 5 × 10^–8^) single nucleotide polymorphism (SNP) was identified as a CypA eQTL; eight SNPs were MMP9 eQTLs; one SNP as a CypA pQTLs and three SNPs as MMP9 pQTLs, in two recent meta-analyses described above. Details of these SNPs are provided in Supplementary Table A below. SNP-outcome estimates for all SNPs were extracted from the Alzheimer’s disease GWAS described above. No SNPs were excluded due to low minor allele frequencies (< 1%) and all studies included in the analyses were coded on the forward strand, thus, no palindromic SNPs were excluded from analyses*.* In an MR analysis, the effect of a SNP on exposure and an outcome must be harmonised to be relative to the same allele. SNPs for the exposure were coded so that the effect allele was always the ‘increasing allele’ (i.e. increasing CypA and MMP9 eQTLs), and the alleles were harmonized so that the effect on the outcome corresponded to the same allele as the exposure.

All SNP-exposure estimates are in standard deviation (SD) units. SNP-outcome estimates are in units of log odds ratios (ORs) for AD. Coefficients were combined using an inverse-variance-weighted (IVW) approach to give an overall estimate of the causal effect across all SNPs included for each eQTL and pQTL. The estimator is a Wald ratio and is equivalent to a weighted regression of the SNP-outcome coefficients on the SNP-exposure coefficients with the intercept constrained to zero. For causal effects of CypA and MMP9 eQTLs and pQTLs on Alzheimer’s disease, results were exponentiated to ORs, thus, causal effect estimates are interpreted as the odds of AD per standard deviation increase in CypA and MMP9 eQTLs or pQTLs.

### UK Biobank polygenic risk score analysis

#### Data

Secondly, we examined whether *APOE4* (C allele of rs429358) and polygenic risk scores for CypA and MMP9 had a causal effect on (i) Alzheimer’s disease-by-proxy (parental dementia) and (ii) continuous markers of cognitive function in the UK Biobank: visual memory (n = 336,679), reaction time (n = 335,019) and fluid intelligence score (n = 120,109). Full details of the UK Biobank and the cognitive assessments are in the online supplement.

#### Statistical analysis in UK Biobank

The current study sample was restricted to those of “White British” origin, who were unrelated, did not report being adopted, and with no missing data for all covariables. Weighted polygenic risk scores were generated for CypA and MMP9 eQTLs and pQTLs (i.e. four different polygenic risk scores), for each participant with genetic data. They included all SNPs associated with CypA and MMP9 eQTLs and pQTLs at genome-wide significance (p ≤ 5 × 10^–8^), clumped at R^2^ = 0.001 and a 10,000 kb window (GWAS refs). Polygenic risk scores were calculated using PLINK (version 2.0). Each score was calculated from the effect size-weighted sum of associated alleles within each participant.

Fluid intelligence scores were approximately normally distributed. Reaction times and visual memory scores were natural log transformed to approximate a normal distribution. A constant of + 1 was added to all visual memory scores to ensure no zeros were log transformed. Parental AD was coded as 0, 1 or 2 parents with AD. Thus, casual effects of the polygenic risk scores on all continuous outcomes were estimated using linear regression models and ordinal logistic regression models were used when parental AD was the outcome. All regression models were adjusted for age and sex. As higher values for reaction time and visual memory (incorrect pair matches) represent poorer performance, we reversed fluid intelligence scores so that they were in a consistent direction with the other continuous outcomes (i.e. higher fluid intelligence scores represent poorer performance). We first conducted these analyses on the whole UK Biobank sample. We then examined these effects within age-stratified tertiles to interrogate potential age-dependent effects. The age-stratified analysis was conducted because, on average, the UK Biobank population is relatively young with respect to AD diagnoses, and if a proportion of the sample have underlying AD pathology, we would anticipate this proportion to be greatest in the oldest tertile. Thus, it is plausible that we may only observe effects of the polygenic risk scores on cognitive impairment indicators in the population which have underlying AD pathology (i.e. the middle to oldest age tertiles). Finally, we examined whether any effects of the polygenic risk score on cognitive impairment indicators were modified by APOE4 carrier status (zero, one or two C alleles for SNP rs429358).

## Results

We found little evidence to suggest *APOE4* has a causal effect on circulating CypA or MMP9 eQTLs or pQTLs using two-sample Mendelian randomization. Confidence intervals were wide and could not exclude a meaningful effect in either direction (Supplementary Table B of the supplement). There was little evidence that CypA or MMP9 eQTLs or pQTLs affected the odds of Alzheimer’s disease, and these estimates were very precise (Table 1 below).

**Table 1:** Causal effects of CypA and MMP9 eQTLs and pQTLs on risk of Alzheimer’s disease.

	N SNPs	F statistics	OR (95% CI)	p
CypA eQTLs	1	775.34	1.00 (0.99–1.01)	0.80
MMP9 eQTLs	8	116.45	1.00 (0.98–1.01)	0.45
CypA pQTLs	1	487.22	1.00 (0.99–1.01)	0.82
MMP9 pQTLs	3	64.04	1.00 (0.98–1.01)	0.51

Causal effect estimates are interpreted as the odds of Alzheimer’s disease per standard deviation increase in CypA and MMP9 eQTLs or pQTLs.

**Figure 1:**
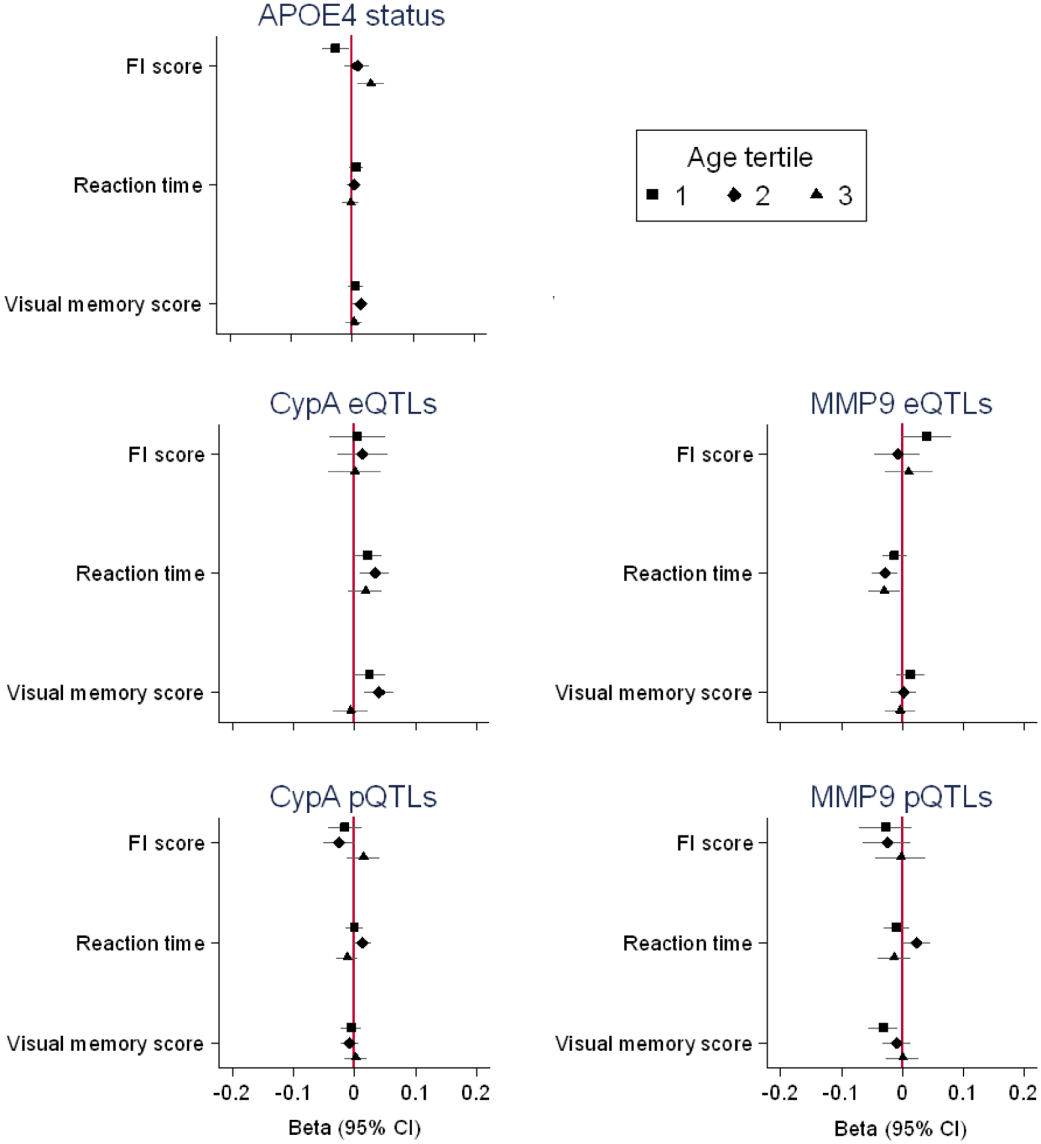
Causal effects of APOE4 carrier status (carrier vs non-carrier) and CypA and MMP9 eQTL and pQTL PRSs on the continuous cognitive function outcomes in the UK Biobank, by age tertiles. All outcomes are in standard deviation units and higher values represent poorer performance.

**Figure 2:**
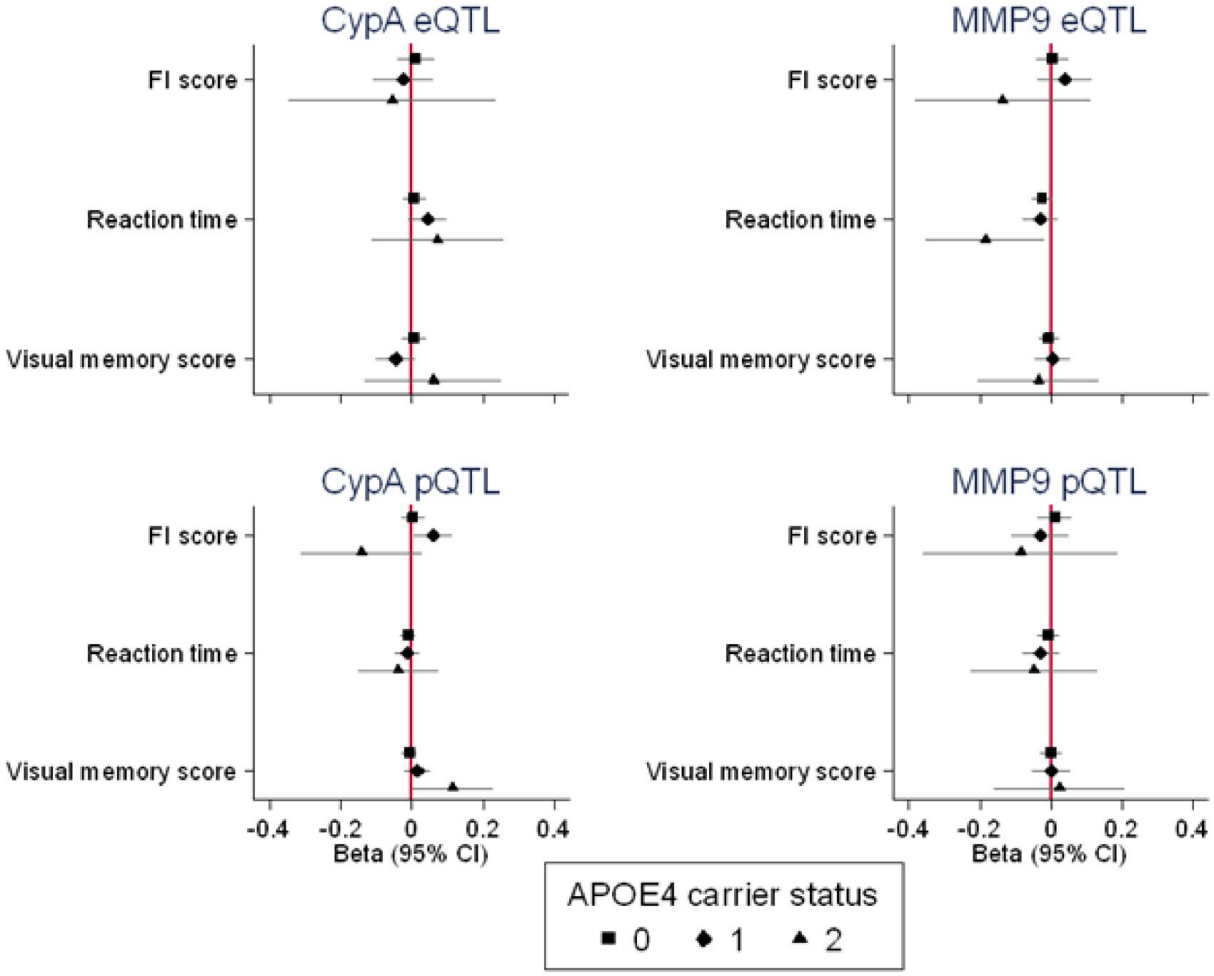
Causal effects of CypA and MMP9 eQTL and pQTL PRSs on the continuous cognitive function outcomes in the UK Biobank (fluid intelligence score, reaction time and visual memory) in the oldest tertile, by APOE4 carrier status. All outcomes are in standard deviation units and higher values represent poorer performance.

In individual-level analyses in the UK Biobank, *APOE4* carrier status was strongly associated with odds of reporting one or both parents to have dementia (Supplementary Table C of the supplement) and there was evidence of an age-dependent effect on fluid intelligence scores (scores worsening with age, Fig. 1). *APOE4* carrier status had no notable causal effect on visual memory scores or reaction times (Fig. 1). Overall, there was no consistent evidence to suggest a causal effect of CypA or MMP9 eQTL or pQTL PRSs on odds of reporting one or both parents to have dementia (Supplementary Table C of supplement) or on any of the continuous markers of cognitive function (Fig. 1). Higher CypA eQTL and pQTL PRSs were associated consistently with faster reaction times in only the middle age tertile. A higher MMP9 eQTL PRS was associated with lower fluid intelligence scores in the youngest tertile only, and with slower reaction times in the middle and oldest age tertiles. Conversely, a higher MMP9 pQTL PRS was not associated with fluid intelligence scores in any age tertile and was associated with faster reaction times in only the middle tertile. Finally, there was little consistent evidence to suggest that causal effects of CypA and MMP9 eQTLs and pQTLs on the continuous cognitive function markers were modified by APOE4 carrier status (Fig. 2). In the oldest age tertile, greater MMP9 eQTL polygenic risk scores were unexpectedly associated with better fluid intelligence scores and reaction times in homozygous *APOE4* carriers, and greater CypA pQTL polygenic risk scores were associated with better fluid intelligence scores, but worse visual memory in homozygous *APOE4* carriers. It is worth noting that these findings were comparable in the whole sample (rather than restricting to the oldest age tertile, results not shown).

## Discussion

We found very little genomic support for CypA as a promising therapeutic target for cognitive impairment in human *APOE4* carriers. Causal effect estimates from the Mendelian randomization analyses were very precisely null, and there was no consistent evidence from the individual analyses on continuous cognitive function markers in the UK Biobank. This casts doubt on whether they are likely to represent effective drug targets for cognitive impairment in human APOE4 carriers. Although statistical power is lower in the UK Biobank analysis compared to the Mendelian randomization analyses, any plausible effects are likely to be extremely small and not clinically meaningful given the confidence intervals estimated. The analyses presented here were conducted on the largest available samples in humans, and we were able to examine a series of related outcomes.

As previously mentioned, recent studies have reported that blockade of the CypA–MMP9 pathway in *APOE4* knock-in mice restores blood brain barrier integrity and subsequently normalises neuronal and synaptic function^2^. Conversely, another recent mouse study reported that whilst while MMP9 inhibition improved specific neurobehavioral deficits associated with AD (such as anxiety and social recognition memory), modulation of MMP9 did not alter spatial learning and memory or Aβ tissue levels in AD animals, suggesting that further work is necessary to understand the nature of the relationship between MMP9 activity and neurological dysfunction in mice. In humans, previous studies of post-mortem cerebrovasculature and of pre-mortem CSF and plasma are conflicting. Some have reported elevated MMP9 and/or CypA in AD cases versus controls, and in moderate to severe AD vs mild AD^11–15^. Additionally, in those studies that stratified on APOE genotype, the highest levels of MMP9 were generally observed in APOE4 carriers. Conversely, other studies have reported lower CSF MMP9 in AD cases compared to controls^16^, and in vascular dementia patients compared to Alzheimer’s patients and controls^17^. Other case–control studies have reported no differences observed in baseline plasma MMP levels between MCI-AD patients and control subjects, and that baseline levels of MMPs do not correlate with longitudinal changes in CSF biomarkers^15^. It is worth noting that it is not possible to establish the direction of causation in these case–control studies, thus it is plausible that MMP9 and CypA levels are altered as a result of advancing AD pathology, as opposed to causing or contributing to it.

There are some limitations to these results. Firstly, CypA and MMP9 eQTLs and pQTLs are from blood and not specifically brain tissue. However, recent evidence^9^ shows brain eQTLs from different brain regions to have very low correlations; indeed some brain regions correlate more strongly with blood and kidney eQTLs than they do with other brain regions. Thus, using eQTLs from brain tissue is not necessarily a useful way to overcome that limitation (as it largely depends on the region analysed) and they are also underpowered (as fewer brain samples exist compared to other tissue samples). It is also worth noting that drugs used to inhibit CypA accumulate in brain microvessels, but do not cross the blood–brain barrier, suggesting blood may be a relevant tissue^2^. Secondly, the UK Biobank population is relatively young with respect to the Alzheimer’s disease course, and we may have been able to detect causal effects in a much older population. That said, there was no evidence of causal effects in the Mendelian randomization analyses which used the largest case–control Alzheimer’s disease GWAS as the outcome, and individual-level results from the UK Biobank looked very similar when restricted to the oldest tertile (age 63–72 years), compared to the youngest and middle age tertile or the whole sample. Finally, it is worth noting that pleiotropy is an unlikely explanation for these findings, as it is likely to bias away from the null. These results are a useful additional source of evidence about the relationship between CypA and MMP9 and Alzheimer’s disease risk, and they should be considered when appraising the likelihood that intervening on these targets will succeed in reducing Alzheimer’s disease risk.

## Supplementary Information

Supplementary Information.
