# Supplementary material for: Little genomic support for Cyclophilin A-matrix metalloproteinase-9 pathway as a therapeutic target for cognitive impairment in APOE4 carriers
Source: Sci Rep. 2022 Jan 20;12:1057. doi: 10.1038/s41598-022-05225-8 (PMC8776806; doi:10.1038/s41598-022-05225-8)
Supplement: Supplementary file 1 — Supplementary Information. [file 41598_2022_5225_MOESM1_ESM.docx]

**Supplemental material

“Little genomic support for cyclophilin A-matrix metalloproteinase-9 pathway as a therapeutic target for cognitive impairment in *APOE4* carriers”**

Instruments used for MR analysis…………………………………………………………………………………..2

UK Biobank data …………………...……………………………………………………………………………………….3

Mendelian Randomization results…………………………………………………………………………………..4

UK Biobank results – parental dementia………………………………………………………………………….4

**Table A: Instruments for CypA and MMP9 eQTLs and pQTLs**

| **Trait** | **RSID** | **Effect allele** | **Other Allele** | **Effect allele frequency** | **Beta** | **Standard error** | **P** |
| --- | --- | --- | --- | --- | --- | --- | --- |
| **CypA eQTL** | rs6463247 | T | C | 0.816911 | 0.417215 | 0.0149835 | 1.2388e-170 |
| **MMP9 eQTLs** | rs3731827 | C | T | 0.418566 | 0.096111 | 0.012037 | 1.41E-15 |
|  | rs7613595 | C | A | 0.23545 | 0.109595 | 0.013998 | 4.89E-15 |
|  | rs149110519 | T | C | 0.052616 | -0.16455 | 0.026622 | 6.37E-10 |
|  | rs56388170 | T | G | 0.289189 | -0.14 | 0.013074 | 9.38E-27 |
|  | rs149007767 | T | C | 0.149708 | -0.14688 | 0.016636 | 1.06E-18 |
|  | rs4065321 | T | C | 0.539612 | 0.077578 | 0.011923 | 7.69E-11 |
|  | rs13925 | A | G | 0.148395 | 0.353837 | 0.016475 | 2.56E-102 |
|  | rs6073969 | T | C | 0.934435 | 0.203758 | 0.023984 | 1.97E-17 |
| **CypA pQTL** | rs62143198 | A | G | 0.21453 | 0.6335 | 0.0287 | 2.86E-108 |
| **MMP9 pQTLs** | rs398076 | A | G | 0.41095 | 0.156 | 0.0255 | 4.75E-10 |
|  | rs62143194 | C | G | 0.77636 | -0.1974 | 0.0306 | 5.56E-11 |
|  | rs2250889 | C | G | 0.95338 | -0.6221 | 0.0585 | 1.03E-26 |

**UK Biobank data**The UK Biobank is a large-scale population-based study that recruited 502,616 individuals, aged 40-69 years old, between 2006-2010. The study was established to enable investigations of genetic and nongenetic determinants of diseases of middle and old age ^6 7^. The UK Biobank was approved by the North West Research Ethics committee. Genotyping was completed for all participants at baseline. Participants also reported whether their mother and/or father had dementia. At the initial visit, reaction time, visual memory and fluid intelligence scores were assessed. Reaction time was assessed using 12 rounds of the card-game 'Snap’. The participant was shown two cards at a time; if both cards were the same, they pressed a button-box that was on the table in front of them as quickly as possible.  Thus, higher values equal poorer performance. Visual memory was assessed using incorrect matches on a pairs matching test. Participants were asked to memorise the position of as many matching pairs of cards as possible. The cards were then turned face down on the screen and the participant was asked to touch as many pairs as possible in the fewest tries. Incorrect matches were counted, thus higher values equal poorer performance. Fluid intelligence was examined in a subsample of participants, by answering a series of questions designed to assess their capacity to solve problems that require logic and reasoning ability, independently of acquired knowledge. The participant had 2 minutes to complete as many questions as possible from the test, and higher scores represent better performance.

**Results**

**Mendelian randomization analyses**

**Table B: Association of APOE (rs429358) with CypA and MMP9 eQTLs and pQTLs**

|  | **Beta (95% CI)** | **P** |
| --- | --- | --- |
| **CypA eQTLs** | -0.01 (-0.05 to 0.03) | 0.62 |
| **MMP9 eQTLs** | 0.001 (-0.04 to 0.04) | 0.97 |
| **CypA pQTLs** | 0.02 (-0.04 to 0.08) | 0.55 |
| **MMP9 pQTLs** | 0.003 (-0.06 to 0.06) | 0.93 |

Causal effect estimates are interpreted as the average difference in CypA and MMP9 eQTLs and pQTLs per risk increasing allele

**UK Biobank results

Table C: Causal effects of APOE4 and polygenic risk scores for CypA and MMP9 eQTLs and pQTLs with odds of reporting one or both parents to have dementia (n=328,509)**

|  | **AD-by-proxy** | |
| --- | --- | --- |
|  | **OR (95% CI)** | **P** |
| **APOE4** | 25.77 (23.08 to 28.77) | <0.0001 |
| **CypA eQTLs** | 1.00 (0.96 to 1.05) | 0.89 |
| **MMP9 eQTLs** | 1.03 (0.99 to 1.07) | 0.22 |
| **CypA pQTLs** | 1.01 (0.98 to 1.04) | 0.51 |
| **MMP9 pQTLs** | 0.99 (0.95 to 1.03) | 0.64 |

Causal effect estimates are interpreted as the odds of reporting one or both parents as having dementia per risk increasing allele of APOE4, and per standard deviation increase in CypA and MMP9 eQTLs or pQTLs.

**References**

1 Montagne, A. *et al.* APOE4 leads to blood-brain barrier dysfunction predicting cognitive decline. *Nature* **581**, 71-76, doi:10.1038/s41586-020-2247-3 (2020).

2 Bell, R. D. *et al.* Apolipoprotein E controls cerebrovascular integrity via cyclophilin A. *Nature* **485**, 512-516, doi:10.1038/nature11087 (2012).

3 King, E. A., Davis, J. W. & Degner, J. F. Are drug targets with genetic support twice as likely to be approved? Revised estimates of the impact of genetic support for drug mechanisms on the probability of drug approval. *PLoS Genet* **15**, e1008489, doi:10.1371/journal.pgen.1008489 (2019).

4 Davey Smith, G. & Hemani, G. Mendelian randomization: genetic anchors for causal inference in epidemiological studies. *Hum Mol Genet* **23**, R89-98, doi:10.1093/hmg/ddu328 (2014).

5 Babenko, V. N. *et al.* Haplotype analysis of APOE intragenic SNPs. *BMC Neurosci* **19**, 16, doi:10.1186/s12868-018-0413-4 (2018).

6 Võsa, U. *et al.* Unraveling the polygenic architecture of complex traits using blood eQTL metaanalysis. *bioRxiv*, 447367, doi:10.1101/447367 (2018).

7 Zheng, J. *et al.* Phenome-wide Mendelian randomization mapping the influence of the plasma proteome on complex diseases. *Nat Genet* **52**, 1122-1131, doi:10.1038/s41588-020-0682-6 (2020).

8 Jansen, I. E. *et al.* Genome-wide meta-analysis identifies new loci and functional pathways influencing Alzheimer's disease risk. *Nat Genet* **51**, 404-413, doi:10.1038/s41588-018-0311-9 (2019).

9 de Klein, N. *et al.* Brain expression quantitative trait locus and network analysis reveals downstream effects and putative drivers for brain-related diseases. *bioRxiv*, 2021.2003.2001.433439, doi:10.1101/2021.03.01.433439 (2021).

10 Burgess, S., Thompson, S. G. & Collaboration, C. C. G. Avoiding bias from weak instruments in Mendelian randomization studies. *Int J Epidemiol* **40**, 755-764, doi:10.1093/ije/dyr036 (2011).

11 Hemani G. *et al*. MR-Base: a platform for systematic causal inference across the phenome using billions of genetic associations. *bioRxiv* (2017).
